# Triggering an unexpected earthquake in an uncoupled subduction zone

**DOI:** 10.1126/sciadv.abf7590

**Published:** 2021-03-24

**Authors:** Matthew W. Herman, Kevin P. Furlong

**Affiliations:** 1 Department of Geological Sciences, California State University, Bakersfield, Bakersfield, CA, USA.; 2 Department of Geosciences, Pennsylvania State University, University Park, PA, USA.

## Abstract

In the 1970s, the Shumagin Islands region of the Alaska subduction zone was identified as a seismic gap expected to host a future great [moment magnitude (*M*_w_) ≥8.0] earthquake. More recent geodetic data indicate that this region is weakly coupled, and the geologic record shows little evidence of past large events. From July to October 2020, a series of earthquakes occurred in this region, raising the possibility of greater coupling. The initial *M*_w_ 7.8 thrust faulting earthquake straddled the eastern edge of the Shumagin Gap and was followed by an *M*_w_ 7.6 strike-slip earthquake within the Shumagin Gap. Stress modeling indicates that this strike-slip earthquake is in fact favored if the Shumagin Gap has low coupling, whereas a highly coupled Shumagin Gap inhibits that type and location of earthquake. The initial thrust earthquake and its afterslip enhanced the strike-slip loading within the subducting slab, helping to trigger the October event.

## Teaser

Two large earthquakes in the Aleutian subduction zone provide unexpected evidence for low plate interface coupling.

## INTRODUCTION

**Fig. 1:**
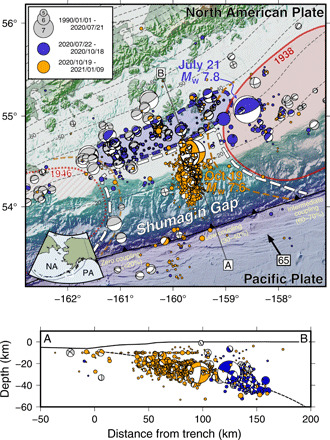
Tectonic setting of the 2020 Alaska-Aleutian subduction zone earthquake sequence. Here, the Pacific (PA) plate subducts under the North American (NA) plate at 65 mm/year (*45*). The 10-km depth contours of the subducting slab are indicated by dashed lines (*43*). Circles and focal mechanisms represent earthquakes from the USGS Comprehensive Catalog, scaled by magnitude and colored by date. Red shaded areas are rupture zones of previous great megathrust earthquakes, inferred from aftershock zones. Geodetic interseismic coupling is indicated along the subduction trench (*8*,*9*). The Shumagin Gap area outlined by the dashed white line has little to no mechanical coupling but is pseudocoupled (hence its low partial geodetic coupling). The shaded blue area down-dip of the Shumagin Gap is the aftershock/afterslip zone of the 21 July earthquake. The dark orange dashed line indicates the expected extent of high pseudocoupling [~80% according to (*21*)] surrounding the coupled parts of the interface. The area of the 1946 earthquake appears geodetically uncoupled, so we indicate the uncertain plate interface behavior in this region with hatched fill. Cross section A-B shows earthquakes and their focal mechanisms projected in side view. These indicate the dominance of deeper thrust faulting plate interface events from July to October, followed by mostly strike-slip events along the top of the slab since 19 October.

Megathrust earthquakes in subduction zones are generally assumed to occur within zones of high plate interface coupling (“asperities”). These earthquakes reflect the recovery of elastic strain accumulated in the plates through this coupling. During the development of plate tectonics, a working model for earthquake potential within subduction zones was proposed in which the likelihood for upcoming great earthquakes along the margin could be simply related to the time since the last great earthquake at that location—the “seismic gap theory” (*1*, *2*). Under that model, the Alaskan-Aleutian subduction zone in the vicinity of the Shumagin Islands was identified as a region with no recent great [moment magnitude (*M*_w_) 8+] earthquakes and therefore having a high potential to host a great earthquake (*2*, *3*). This section of the plate interface from 158° to 161°W was termed the “Shumagin Gap” (Fig. 1). Although the seismic gap theory has been challenged (*4*), the question of whether the Shumagin Gap represents a major earthquake hazard has persisted.

More recently, geodetic data collected near the Shumagin Islands have been interpreted to indicate variable plate coupling along the subduction zone. These observations indicate that the region to the east of the Shumagin Gap (which hosted a great earthquake in 1938) is currently strongly coupled and accumulating slip deficit. In the Shumagin Gap itself, early triangulation surveys (*5*, *6*) and current Global Positioning System (GPS) observations (*7*–*9*) indicate that the plates in that region are not well coupled, except for a possibility of coupling near the trench, which is poorly constrained by the onshore data. Such low coupling implies that the plate interface is not likely to accumulate sufficient stress to generate a great megathrust earthquake. The absence of great earthquakes within the Shumagin Gap is also supported by the results of paleoseismic studies (*10*) that do not find any evidence of either substantial vertical motions (which occur during the earthquake cycle along coupled margins) or tsunami deposits generated by large, local earthquakes. The combined seismic, geodetic, and geologic evidence strongly suggests low plate interface coupling in the Shumagin Gap. In contrast, despite geodetic observations indicating poor coupling along the subduction plate interface west of 161°W, this region hosted a great (*M*_w_ 8.2 to 8.5) earthquake in 1946 (*11*, *12*). There is also geologic evidence for tsunamigenic earthquakes every ~300 years, although these tsunami records may be related to the locked patch associated with the 1957 earthquake further to the southwest (*13*). These records of great earthquakes suggest that there may be some coupling along the plate interface west of the Shumagin Gap, but the exact distribution of coupling in this region remains uncertain (Fig. 1). On the basis of the overall evidence from geodetic and geologic studies, the general view of the Shumagin Gap in early 2020 was that it was poorly coupled and unlikely to host a great earthquake.

A recent sequence of earthquakes along the Alaska-Aleutian subduction zone, which occurred initially adjacent to, but then encroached into the Shumagin Gap, again raises the idea that the Shumagin Gap may be able to host a great earthquake. This sequence highlights the issue of which type of seismic gap is the Shumagin Gap—uncoupled and creeping, or coupled and (akin to Cascadia, perhaps) generally aseismic between great earthquakes. The initial earthquake in the 2020 sequence was a subduction interface earthquake (*14*, *15*). It occurred within the inferred rupture zone of the 1938 great earthquake and so was not unexpected. In contrast, the subsequent “gap” earthquake in October 2020 was a strike-slip earthquake within the subducting Pacific Plate. In this study, our modeling of the stresses along this plate boundary before and during this earthquake sequence indicates that the occurrence of the strike-slip event is favored if the Shumagin Gap has low plate interface coupling and slips relatively aseismically. Note that this aseismic characteristic specifically refers to subduction megathrust events. As we explore below, the plates within the uncoupled Shumagin Gap are sufficiently stressed to host large, but not subduction interface, earthquakes.

The July to October 2020 earthquake sequence began with a major (*M*_w_ 7.8) subduction interface earthquake on 21 July (Fig. 1). This event generated a local tsunami with wave amplitudes of up to ~0.5 m at the Sand Point tide gauge north of the Shumagin Islands and ~15 cm at the Southeast Chirikof Deep-ocean Assessment and Reporting of Tsunamis (DART) buoy in the Pacific Ocean. The rupture began in a region of intermediate geodetic interplate coupling (60 to 70%), just west of the region of highest coupling along the Aleutian arc [>90%; (*8*, *9*)]. The history of earthquakes in this region also indicates substantial interplate coupling; the July 2020 event occurred in a similar location to an *M*_w_ ~7.4 thrust earthquake in 1917 (*16*), which last ruptured as part of a great earthquake in 1938 (*17*, *18*). The seismically derived rupture model for the July earthquake places most of the coseismic slip within the highly coupled region, indicating that rapid slip occurred in this area, producing considerable seismic radiation (*19*). This is consistent with back-projection results showing seismic radiation being generated primarily from the region around the epicenter (*20*). Models incorporating static (*15*) or high-rate (*14*) GPS observations into their slip analysis image additional coseismic slip extending westward beneath the Shumagin Islands; although this coseismic slip is substantial, it appears to have produced less intense seismic radiation. On the basis of the location differences between the seismically derived rupture pattern and the geodetically derived coseismic slip, it appears that the coseismic rupture migrated westward into a region of “pseudocoupling” between the coupled zone and the Shumagin Gap. Pseudocoupling refers to a region of the plate interface that is mechanically uncoupled but still deformationally acts as coupled due to its proximity to nearby mechanically coupled areas (*21*). Postseismic afterslip and aftershocks from the *M*_w_ 7.8 earthquake occurred on the plate interface surrounding the coseismic rupture zone starting immediately after the mainshock rupture, as is typically expected following a megathrust earthquake [United States Geological Survey (USGS) Comprehensive Catalog] (*22*). Virtually, all of these aftershocks are thrust faulting events occurring on or near the plate interface. This postseismic activity extended to the southwest along the plate interface (*14*), including along the plate boundary zone down-dip of the uncoupled section of the Shumagin Gap.

On 19 October 2020, a major (*M*_w_ 7.6) intraplate strike-slip earthquake occurred 80 km southwest of the July epicenter, within the region identified in geodetic inversions to have low (30 to 40%) interseismic coupling (*8*, *9*). This level of apparent coupling is consistent with the interpretation that the plate boundary in the Shumagin region is uncoupled and freely slipping but still accumulating slip deficit (thus appearing partially coupled) due to its proximity to a locked zone farther east (*21*). An unexpected aspect of this event is that despite being strike-slip, it also produced a tsunami with wave heights comparable to or larger than the tsunami generated by the July megathrust event: ~1 m at the Sand Point tide gauge and ~15 cm at the Southeast Chirikof DART buoy. In contrast to the earlier series of shallowly dipping, thrust faulting subduction zone earthquakes, this event ruptured as a more steeply dipping, nearly trench-perpendicular strike-slip fault within the subducting Pacific plate, immediately below the megathrust (Fig. 1). The October event and all of its aftershocks are high-angle, right-lateral, strike-slip earthquakes defining an approximate north-south (N-S) fault zone. Although *M*w 7.6+ earthquakes are not rare, as three to four of them occur annually around the world, strike-slip earthquakes of this size are less common. The October *M*_w_ 7.6 event is one of the 10 largest intraplate strike-slip earthquakes to have occurred over the past 40 years and the only one to occur within the shallow part of a subduction zone. Most strike-slip earthquakes of comparable size rupture major plate boundary structures or in the outer rise.

The patterns of earthquakes and afterslip throughout this sequence suggest that (i) strain recovery associated with megathrust slip within pseudocoupled regions may not always produce a seismological signature even when occurring at earthquake strain rates, but such aseismic slip can be observed geodetically in the near field; (ii) although plate interfaces may be relatively stress free in uncoupled regions, the plate interiors in the transition zone between low and high coupling can become sufficiently stressed to host moderate-to-large earthquakes with faulting styles atypical for subduction zones; (iii) such an atypical intraplate earthquake can be triggered in response to stress changes caused by nearby, more common subduction interface thrust earthquakes; and (iv) likewise, aftershocks and afterslip can occur even in regions down-dip of a mostly decoupled plate interface when driven by adjacent earthquake slip.

We use a suite of numerical models to address these hypotheses by evaluating the spatial and temporal stress evolution in the vicinity of the Shumagin Gap. Specifically, we investigate the conditions that could lead the July plate interface thrust faulting earthquake to trigger the October intraplate strike-slip earthquake. This sequence of two major (*M*_w_ 7.8 and 7.6) earthquakes within the Alaska-Aleutian subduction zone in the vicinity of the Shumagin Islands seismic gap thus provides a means to assess models and interpretations of (i) the spatial distribution of plate boundary coupling in this seismicity gap; (ii) the development of intraplate stress conditions that can generate large, nonsubduction interface earthquakes; and (iii) implications that non–plate interface earthquake sources can be damaging (tsunamigenic, strong ground shaking, etc.) within otherwise low earthquake-hazard subduction zone segments.

## RESULTS

**Fig. 2:**
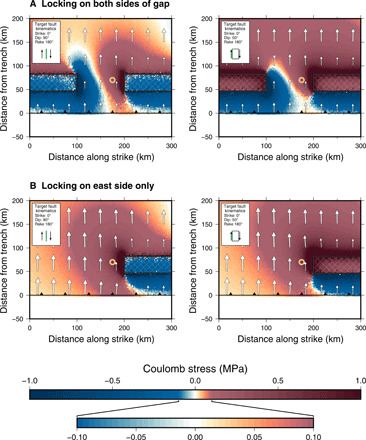
Modeled stresses in the slab near the Shumagin Gap before the 2020 earthquake sequence. (**A**) Coulomb stresses along the surface of the subducting slab, resolved onto trench-perpendicular, right-lateral, strike-slip faults. This model has two locked patches (indicated by black rectangles with cross-hatching) and a 100-km-long freely sliding zone between them. Red areas are where right-lateral slip is promoted, and blue areas are where that slip is inhibited. The stress magnitudes correspond to 82 years of interseismic loading. Arrows represent the displacements at the top of the slab relative to the displacement in the center of the locked zone and show the shearing that develops across the edges of the uncoupled gap. (**B**) Coulomb stresses along the surface of the subducting slab for a model with only one locked patch on the east side of the uncoupled zone. Without a locked zone in the west, the stresses in the slab under the uncoupled regions favor exclusively the right-lateral faulting.

To understand the deformation and stress conditions in the slab beneath the Shumagin Gap region resulting from the coupling distribution on the plate interface, we develop a generalized numerical subduction zone model. The model has a planar slab and locked patches on the plate interface representing the 1938 (and potentially 1946) asperities separated by a zone of free slip representing an uncoupled Shumagin Gap (see Materials and Methods). Results from these models suggest that the transition from high coupling in the 1938 rupture zone to low coupling in the Shumagin Gap is needed to explain the faulting characteristics of the October large strike-slip earthquake. The intraslab stress field produced on the east side of the Shumagin Gap uncoupled zone during the 82 years of loading since the last great earthquake in the region strongly promotes right-lateral strike-slip failure on trench-perpendicular faults (Fig. 2). This is true for either shallowly dipping structures (comparable to the October 2020 mainshock moment tensor) or steeply dipping faults (similar to many of the October 2020 aftershock focal mechanisms). The cause of this stress is straightforward: The coupling between the upper plate and the slab in the locked patch applies a trenchward-directed traction to the slab, reducing its down-dip motion. In contrast, the low coupling in the gap means that the slab can move more freely down-dip. This spatial variation in displacements produces a large-magnitude, right-lateral shear stress, which is absent if the plate interface is more uniformly coupled along strike. In a similar fashion, these induced stresses from 82 years of plate motions on the west side of the Shumagin Gap would favor left-lateral strike-slip faulting, assuming that the area of the 1946 rupture zone is coupled (Fig. 2A). If the west side of the Shumagin Gap is uncoupled, as geodetic observations suggest, then right-lateral strike-slip faulting is favored throughout the slab beneath the entire uncoupled region (Fig. 2B). If the Shumagin Gap region were more strongly coupled, then our model results suggest that the intraslab stresses would inhibit the type of strike-slip faulting that occurred in October (fig. S1).

**Fig. 3:**
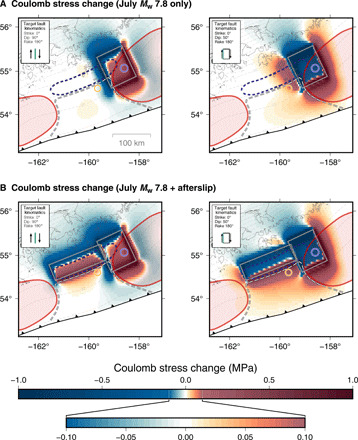
Modeled stress changes in the slab during the July to October 2020 earthquake sequence. (**A**) Coulomb stress changes produced by the July *M*_w_ 7.8 earthquake (slip area indicated by the rectangle) in the slab, resolved onto right-lateral strike-slip faults with the geometry of the October earthquake. If the target fault is steeply dipping (left), then the stress changes at the location of the October *M*_w_ 7.6 event (orange circle) are near zero to slightly negative. In contrast, if the target fault is more shallowly (50°) dipping (right), then the July event positively loads the *M*_w_ 7.6 hypocentral region. (**B**) For either target fault geometry, the subsequent afterslip and aftershocks down-dip of the Shumagin Gap load the *M*_w_ 7.6 hypocenter.

In addition to the stresses accumulated in the slab over the interseismic period, slip during the July earthquake and its afterslip (including aftershocks) modify the stress field. Using elastic half-space models, we estimate these stress changes along the top of the subducting Pacific slab, focusing particularly on the hypocentral region of the October *M*_w_ 7.6 event (see Materials and Methods and Fig. 3). How the July *M*_w_ 7.8 mainshock loads the *M*_w_ 7.6 hypocenter depends on the dip of the activated October fault (Fig. 3A). If it is steeply dipping, then the Coulomb stress changes produced by the *M*_w_ 7.8 are small to slightly negative. However, if the fault dips more gently (less than ~65°), as indicated by the moment tensors determined by the USGS and Global Centroid Moment Tensor Project (*23*), then the *M*_w_ 7.8 positively loads the *M*_w_ 7.6 hypocenter. Independent of the dip of the strike-slip fault, the afterslip from the *M*_w_ 7.8 event provides an additional, strong load of the *M*_w_ 7.6 hypocenter, resulting in net positive Coulomb stress changes (Fig. 3B). These results indicate that the long-term stress buildup since the previous great earthquakes produced a stress field favoring the strike-slip event; then, in 2020, the *M*_w_ 7.8 mainshock and subsequent down-dip afterslip enhance that stress field and helped trigger the strike-slip event.

## DISCUSSION

**Fig. 4:**
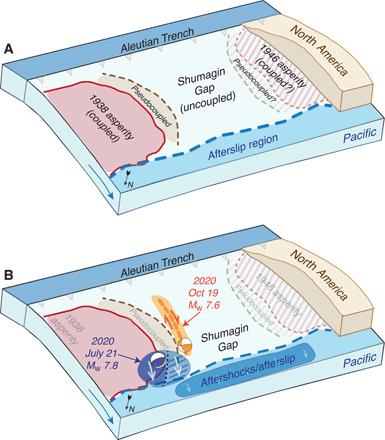
Synoptic view of the plate tectonic setting and plate interface conditions in the Shumagin Gap region. (**A**) Coupling conditions since the mid-20th century earthquakes bounding the gap region. The asperity that ruptured in 1938 is assumed to have relocked, producing both fully coupled and pseudocoupled regions. The presence of a similar asperity on the west side of the Shumagin Gap associated with the 1946 rupture is less certain because geodetic observations indicate low coupling there. The region down-dip of the coupled zones (and extending into the Shumagin Gap) is a transition zone where coseismic slip, aftershocks, and postearthquake afterslip may occur. Within the Shumagin Gap, the transition from coupled to uncoupled next to the 1938 asperity favors right-lateral slip perpendicular to the trench. (**B**) Conditions associated with the 2020 earthquake sequence. The July *M*_w_ 7.8 event (blue) ruptured across the edge of the coupled zone. It produced aftershocks and afterslip in the vicinity of the earthquake and also within the afterslip region down-dip of both the coupled and uncoupled sections of the plate boundary. The coseismic slip, afterslip, and aftershock activity in this down-dip region increased the Coulomb stress within the slab favoring triggering of the October *M*_w_ 7.6 strike-slip event (orange).

Our interpretations of the plate coupling conditions and the sequence of events that led to this multicomponent earthquake sequence are shown in Fig. 4. The conditions on the plate interface—the top of the subducting Pacific Plate—that produced the stress field acting at the time of the July subduction thrust-faulting earthquake include at least one asperity region (where the subducting and overriding plates are coupled and thus moving together) on the east side of the uncoupled Shumagin Gap region (Figs. 2 and 4A). Because of this low coupling, there is free slip between the two plates in the Shumagin Gap, except for the pseudocoupling effects produced by the mechanical continuum between the coupled asperities and the adjacent uncoupled plates (*21*). The subducting slab around this pseudocoupled region accumulates shear stresses as a result of the displacement gradient between the coupled and uncoupled regions (vectors in Fig. 2). Sufficiently distant from that coupled/uncoupled transition (greater than ~100 km), the subducting plate within the interior of the Shumagin Gap does not develop these shear stresses.

In most earthquake systems, there is a transition at depth along a fault where deformation switches from a brittle frictional (stick-slip) process to a stably sliding or ductile deformation regime. Beyond this depth, much of the relative plate motion is continuously accommodated aseismically, and little slip deficit accumulates. It is common in this transition region for both modes of plate boundary slip to occur in response to nearby larger events—a combination of earthquakes (coseismic slip or aftershocks) and relatively rapid aseismic afterslip. We refer to this section of the plate boundary simply as the “afterslip region” (Fig. 4A).

Our stress modeling results indicate that the distribution of asperities (coupled patches) typical in subduction zones produces an area within the asperities and extending into the adjacent pseudocoupled zone with sufficient slip deficit and stress conditions favorable for generating subduction zone thrust-faulting earthquakes (fig. S2) (*21*). The occurrence of the July *M*_w_ 7.8 thrust event and its apparent dual characteristics of seismic (in the coupled region) and aseismic (in the pseudocoupled region) slip is expected for an event on the edge of a well-coupled asperity (Fig. 4B). This event also increases the stress conditions favoring low-angle, plate interface thrust faulting in the region around the rupture, including within the afterslip region shown in Figs. 3 and 4 (fig. S3A). The seismicity behavior of the *M*_w_ 7.8 subduction thrust rupturing into and subsequently triggering aftershocks and afterslip immediately westward from the rupture is consistent with typical plate interface activity. However, what distinguishes this earthquake sequence from a more typical sequence of subduction thrust events is that the aftershocks and afterslip remain within the transition region near the base of the seismogenic zone and do not extend further up-dip along the plate interface in the Shumagin Gap region (Fig. 1) (*14*, *22*). There are no thrust faulting events in either the July or October aftershock sequence that can clearly be interpreted to reflect coseismic slip along the plate interface within the seismogenic zone of the Shumagin Gap. This is expected if the Shumagin Gap is uncoupled, although the lack of seismicity alone would be insufficient to infer low coupling (e.g., the Cascadia subduction zone is currently aseismic but appears to be strongly coupled and has produced great megathrust earthquakes). Given the uncoupled state of the Shumagin Gap, the robust aftershock and afterslip activity down-dip of the base of the seismogenic zone is perhaps unexpected but consistent with the distribution of historical seismicity (Fig. 1) (*24*). This implies that, rheologically, the down-dip edge of the seismogenic zone at 30- to 40-km depth [which may represent a transition in frictional characteristics, e.g., (*25*); a change in plate interface thickness, e.g., (*26*); or the beginning of the brittle-to-ductile transition as defined by thermal models, e.g., (*27*)] in the Shumagin Gap region behaves similarly to the equivalent region down-dip of fully coupled sections of the subduction plate interface. There may be small (<20 km wide), coupled asperities in this down-dip region capable of hosting up to *M*_w_ ~6.5 earthquakes, but any asperities larger than this would likely be visible geodetically. An alternative explanation for the wide along-strike extent of this aftershock activity, despite little stress accumulation in this region (figs. S2 and S3), comes from the pseudocoupling framework (*21*). Slip deficit has accumulated in the Shumagin Gap—as seen in geodetic inversions—which we interpret to be caused by proximity to the locked zone farther east. Once this locked zone or parts of it have been released by an earthquake, this slip deficit can subsequently slip as aftershocks or afterslip.

The *M*_w_ 7.6 earthquake in October 2020 is less expected, rupturing a high-angle (dipping ≥50°) strike-slip fault and located within the subducting Pacific plate (Figs. 1 and 4B). What at first glance might seem to be anomalous behavior—strike-slip faulting in an otherwise aseismic, decoupled subduction zone segment—is produced by the displacement gradient associated with the transition from a fully coupled to uncoupled plate interface (Fig. 2). Even without the occurrence of the July *M*_w_ 7.8 earthquake, the stresses in the slab strongly favor the kinematics of the October event. This intraplate deformation is augmented (post-July earthquake) by down-dip slip in the brittle-to-ductile transition zone through a combination of aftershocks and aseismic afterslip (Fig. 3B). This additional (but smaller magnitude) loading on the October fault appears to have triggered its rupture. We also assess whether similar stress conditions favoring intraplate strike-slip faulting can be produced if there is moderate-to-full coupling in the Shumagin Gap. With strong plate coupling in the Shumagin Gap region (fig. S1), the interseismic stress accumulation within the subducting Pacific plate is different. Coulomb stress analyses of our model results indicate that high-angle strike-slip faulting would be inhibited in this region. As the dip of the strike-slip fault shallows, strike-slip failure of such a structure can become more favorable (Fig. 2); essentially, the strike-slip fault is acting progressively more like a moderate angle thrust fault. In addition, it should be noted that if such Shumagin Gap coupling were by an asperity separated from the 1938 and 1946 asperities, then the eastern part of the Shumagin Gap remains unfavorable for trench-perpendicular, right-lateral strike-slip faulting, even with a dip comparable to what was observed in October 2020. Overall, with a coupled Shumagin Gap, earthquake activity in that region would be more likely expected to be typical thrust faulting on the plate interface or associated plate interface splay faults.

Although the stress field across the coupled-uncoupled boundary at the east edge of the Shumagin Gap can drive right-lateral strike-slip within the subducting Pacific plate, this may not be sufficient to cause an earthquake of such large magnitude without an existing host fault. Large intraplate earthquakes often rupture along preexisting faults that are favorably oriented for slip in the applied stress field [e.g., the 2012 Indian Ocean earthquake (*28*), the 2010–2012 Canterbury earthquake sequence (*29*), and the 2018 Gulf of Alaska earthquake (*30*)]. There is no evidence for an N-S structure near the Shumagin section of the subduction zone that could have hosted the October 2020 earthquake, but the northern Pacific plate farther west does contain fossil N-S–oriented fracture zones from the Kula-Pacific midocean ridge system (*31*). The east-west magnetic anomalies indicative of this extinct midocean ridge system extend across the Shumagin Islands (*9*), so an N-S–oriented fracture zone within the subducting plate near the Shumagin Gap is a plausible candidate for hosting the October earthquake. If such a fracture zone exists, then it is not apparent in the seafloor bathymetry, implying that it is not actively slipping before entering the subduction zone. Alternatively, such a trench-perpendicular fault might have been formed by other tectonic stresses in the slab. For example, the dip of the subducting Pacific plate increases from east to west, which could be associated with trench-parallel tensile stresses along the top of the slab. This geometry might therefore help create N-S faults and also enhance the potential for slip on trench-perpendicular faults like the October event.

The October strike-slip earthquake produced a local tsunami similar to the July thrust faulting event, despite not having much vertical component to its fault slip. We assessed the seafloor vertical displacements that would be associated with these two earthquakes to address this apparent conundrum (fig. S4 and Materials and Methods). The July plate interface thrust faulting earthquake ruptured near the base of the seismogenic zone at 30-km depth, uplifting the seafloor up to ~70 cm. However, the depth of this event likely reduced its vertical displacement magnitudes. The October strike-slip event also can generate substantial vertical seafloor displacements, including ~40 cm of subsidence near the Shumagin Islands and ~50 cm of uplift closer to the trench, as it ruptured up the slab toward the trench. The moderate eastward dip of the fault (~50°) accentuated this seafloor motion compared with a steeply dipping strike-slip fault. Tsunami generation typically requires large vertical seafloor movements to displace the water column. Although this condition most frequently occurs in subduction zones during shallow plate interface thrust faulting earthquakes, any event that vertically displaces a sufficient region of the seafloor can be a tsunami source. Tsunamis can also be generated by large horizontal displacements of steep underwater topography, which might be important for this event or other strike-slip–generated tsunamis [e.g., the 1994 Mindoro, Philippines tsunami (*32*), the 2018 Palu, Sulawesi tsunami (*33*), and the 2018 *M*_w_ 7.9 Gulf of Alaska earthquake (*30*))]. The October 2020 strike-slip earthquake is perhaps an unusual tsunami source, but there is no evidence in the mainshock or the aftershocks for anything other than strike-slip faulting (Fig. 1), and its predicted surface displacements show considerable vertical and horizontal displacements over a moderately large area. Therefore, we infer that the tsunami occurrence is consistent with having been caused by the October strike-slip earthquake.

In the absence of plate interface thrust faulting earthquakes, slip on shallow splay faults in the upper plate can also generate tsunamis (*34*–*36*). Although the plate interface in the Shumagin Gap appears to be uncoupled, our modeling results show that the Pacific plate below the uncoupled zone accumulates stresses (Fig. 2 and fig. S2). Similarly, the upper plate will also be stressed above the uncoupled zone next to a coupled region. We cannot entirely rule out the possibility that the October event triggered slip on these shallow faults coseismically, contributing additional seafloor motion to the tsunami source. If so, this triggered slip occurred relatively aseismically (otherwise we would expect to see some indication of this faulting in the seismic characteristics of the mainshock) and without generating much aftershock activity (otherwise we would expect to see these aftershocks). Although these faults did not slip at the same time as the October event, they could still slip in later tsunamigenic earthquakes, so the question remains: Did the 2020 earthquakes load shallow crustal faults? To evaluate the potential for triggering slip on one of these faults, we analyzed the cumulative stress effects of the 2020 earthquake sequence (including the July *M*_w_ 7.8, its afterslip, and the October *M*_w_ 7.6) on shallow (10 km) thrust faults (fig. S3D). The largest magnitude Coulomb stress changes occur south of the *M*_w_ 7.8 and east of the *M*_w_ 7.6 rupture zones, and shallow thrust faults have been positively loaded elsewhere throughout the Shumagin Gap. Although these splay faults may not be capable of great (*M*_w_ 8+) earthquakes as in a coupled megathrust region, a triggered *M*_w_ 7 to 8 earthquake on one of these faults could produce substantial seafloor displacements and therefore regional tsunamis.

### Summary

The recent sequence of two large earthquakes (and their associated aftershocks) along the Alaska-Aleutian subduction zone provides strong evidence, perhaps paradoxically, for the decoupled nature of the Shumagin Seismic Gap. The sequence of events and, in particular, the triggering of an intraplate strike-slip earthquake within the gap are most consistent when the Shumagin Seismic Gap region is a decoupled segment of the subduction plate boundary, compatible with recent models from GPS data. If the plate interface is coupled in the gap, then it is hard to produce stress conditions within the subducting plate to produce a large (*M*_w_ 7.6) strike-slip earthquake. We also find that although regions such as the Shumagin Gap have a low seismogenic potential for plate interface thrusting, so the seismic and tsunami hazard might be thought to be small, the existence of this decoupled region increases the potential for intraplate strike-slip faulting. Thus, other apparently uncoupled sections of subduction zones (e.g., in northern Peru, the northern Hikurangi zone in New Zealand, or sections of the Central America plate boundary), which otherwise might be thought to have relatively low hazard potential, could still host earthquakes with the potential to produce strong shaking and regional tsunamis. Despite the additional evidence from this earthquake sequence for a decoupled Shumagin Gap, the cause of this low coupling here and in other global subduction zones remains enigmatic.

## MATERIALS AND METHODS

### Pseudocoupling and framework stress model

We use a suite of simple, finite element numerical three-dimensional subduction zone deformation models to investigate the stress conditions in the slab caused by lateral variations in plate interface coupling, based on the models in (*21*). The equations for mechanical equilibrium in these models are solved using the finite element platform GTECTON (*37*). The model domain consists of an upper plate and a planar subducting slab dipping at 25° (fig. S5). We choose this simple geometry to isolate the effects of locking on the intraplate stress field, because the interactions between the rheology of the plate interface, the stresses within the plates, and the geometry of the system over the decades-to-centuries time scale of loading complicate and may otherwise mask the effects of locking [e.g., (*37*)]. The model is sufficiently large to minimize the effects of boundary conditions on the deformation: 1000 km along strike, 400 km from the trench to the back of the upper plate, 200 km from the trench to the ocean side, and 500 km deep. Because we are focusing on the deformation in the shallow, brittle/elastic part of the subduction system, the model is purely elastic with a shear modulus of 40 GPa and Poisson’s ratio of 0.25. Deformation in the model is driven by relative plate motions. The slab moves 1 m down-dip, applied at the top and bottom of the subducting plate, and, in this elastic system, the effects can be scaled to represent any magnitude of plate motion. The back of the upper plate is held fixed, acting as the backstop for the subduction system. Along the dipping plate interface, we allow discontinuous slip by applying slippery node boundary conditions (*38*). We define locked zones on the plate interface as having zero slip by applying a force proportional to the fault slip (i.e., a differential spring force), with spring constant 1 × 10^20^ N/m. Everywhere outside of the locked zones on the plate interface is allowed to slide freely with zero shear tractions.

In these models, by the assigned boundary conditions, locked zones experience no slip between the two plates, but they may be displaced relative to the backstop. Unlocked areas of the plate interface near locked zones are not able to slide at the full relative plate motion because of their continuum mechanical connection to adjacent locked sections. These areas appear coupled or partially coupled, despite the fact that their boundary conditions would allow the full plate motion in the absence of locked zones. Therefore, we refer to these regions as “pseudocoupled.” The pseudocoupling effect diminishes with distance from the locked patch, and the slip deficit in these regions can potentially be recovered coseismically or postseismically (*21*). The locked zones in our models of the Shumagin Gap extend 40 km down-dip, centered at a depth of 30 km (i.e., 64 km from the trench). In our range of models, we vary the along-strike length and distribution of these locked patches to assess the effect of coupling distribution on the deformation field in the slab.

We monitor both the displacement and stress fields along the surface of the slab 1 km below the dipping plate interface. The displacement of the slab surface is plotted as arrows in Fig. 2. To make clear the variations in this displacement field across the model that can generate associated strain and stresses, we show the displacements relative to the displacement in the locked zone. Using the stress tensor at each point in the model, we calculate the Coulomb stress

$$\text{CS}=\tau +{\mu \sigma }_{n}$$

where τ is the shear stress, σ_n_ is the normal stress (positive = dilation), and μ is the effective coefficient of friction (set to 0.5 in this study). Stresses are variously resolved (as appropriate) on trench-perpendicular, right-lateral, strike-slip faults (simulated stress conditions for the October 2020 earthquake) and on thrust faults with the geometry of the plate interface.

### Coseismic Coulomb stress changes

After evaluating the role of long-term plate motions acting on the asperity distribution, we determine the role of the July *M*_w_ 7.8 mainshock and subsequent afterslip and aftershocks in triggering the October *M*_w_ 7.6 earthquake by calculating the Coulomb stress change (*39*, *40*) caused by the subsequent events, using the approach in (*41*). This aspect of the modeling uses an elastic half-space configuration with the same elastic properties as the finite element models described above. In contrast to the interseismic modeling described above, the rheology of the system over the shorter time scales of the earthquake sequence is relatively simple and elastic and thus less affected by the choice of geometry. This allows us to (i) treat the system like an elastic half-space while (ii) incorporating the more representative geometries for the faults and slab. This focused Coulomb stress change modeling uses the equations of (*42*) to compute the deformation around rectangular fault patches with uniform slip.

The July *M*_w_ 7.8 mainshock location and geometry are based on a combination of the USGS, Crowell and Melgar (*14*), and Liu *et al*. (*15*) finite fault models, adjusted slightly to correspond to Slab2 (*43*). The center of the rectangular fault zone is at 158.90°W, 55.10°N, and 32-km depth. The fault has a strike of 244°, a dip of 16°, and a rake of 72°. The down-dip width of the fault is 90 km, and the along-strike length is 70 km. We apply uniform slip of 2.0 m, resulting in an earthquake with a magnitude of 7.8. The aftershock slip zone is based on the distribution of aftershocks and the afterslip inversion (*14*). The center of the afterslip fault zone is at 160.45°W, 54.84°N, and 38-km depth. The fault has a strike of 248°, a dip of 18°, and a rake of 90°. The down-dip width of the afterslip zone is 45 km, and the along-strike length is 140 km. We apply uniform slip of 0.75 m, so the afterslip has a moment magnitude equivalent of 7.4.

Stress changes from the *M*_w_ 7.8 earthquake and afterslip are calculated 1 km below the plate interface as defined by Slab2 (*43*). We then calculate the Coulomb stress change resolved onto faults with the geometry of the October *M*_w_ 7.6 event to determine whether and to what degree these preceding events could trigger the October earthquake. We test the effect of varying this target fault dip from 90° to 50°. We also calculate the Coulomb stress changes on the plate interface for thrust faulting.

### Coseismic surface displacements

To assess the tsunamigenic potential for these earthquakes, we calculate the surface displacements produced by the events (fig. S4). We assume that the fault slip occurred in an elastic half-space with the same elastic parameters as described above and that the ruptures occur on rectangular faults with uniform slip. We use the equations of Okada (*42*) to compute the displacements, using the approach in (*41*). The July *M*_w_ 7.8 fault has the same geometry as in the Coulomb stress change calculation. The October *M*_w_ 7.6 earthquake is centered at 159.66°W, 54.41°N, and 25 km. This depth is shallower than the hypocentral depth to reflect the up-dip propagation of the rupture. The down-dip width of the fault is 20 km, the along-strike length is 70 km, and the fault has 5 m of slip. We test two fault orientations: strike = 355°, dip = 50°, and rake = 175° (the USGS best-fitting fault plane), and strike = 355°, dip = 90°, and rake = 175° (a steeply dipping strike-slip fault).

## SUPPLEMENTARY MATERIALS

Supplementary material for this article is available at http://advances.sciencemag.org/cgi/content/full/7/13/eabf7590/DC1
